# Comparative analysis of first-attack performance in elite women’s volleyball at the 2020 and 2024 Olympic Games

**DOI:** 10.3389/fspor.2025.1658390

**Published:** 2025-10-28

**Authors:** Meng'Juan Wang, Jie Wang

**Affiliations:** School of Athletic Performance, Shanghai University of Sport, Shanghai, China

**Keywords:** women's volleyball, performance analysis, first attack, TACTICS, match analysis

## Abstract

This study compares and analyzes the performance of elite women's volleyball teams in various aspects of the first attack during the 2020 Tokyo Olympics (OG-20) and the 2024 Paris Olympics (OG-24). It reveals the changing trends in the first attack system of elite women's volleyball teams, particularly in receiving serves, setting, and attacking. By combining video analysis, the study identifies the underlying causes of issues within the first attack system and provides an overall understanding of the development trends of the first attack system in elite women's volleyball. The study findings provide a theoretical basis for coaches to develop targeted training programs and optimize tactics, and serve as a reference for elite women's volleyball teams preparing for the 2028 Olympic Games.

## 1 Introduction

Volleyball is a skill-oriented net sport in which athletic performance is assessed using a point-based scoring classification system (1). In this category of competition, offensive and defensive actions contribute to point gains and losses. Athletes enhance their likelihood of victory mainly by reducing defensive errors and increasing offensive scoring. Additionally, variations exist in the point efficiency of different technical and tactical approaches (2).

Since the International Volleyball Federation decided to adopt the “rally-point system” for volleyball matches in 1998, the importance of the first attack has remarkably increased. The success or failure of the first attack directly determines the overall course of the match (3). In volleyball, the “first attack” refers to the first offensive organization following the reception of the opponent's serve, comprising three components: serve reception, set, and attack. With the development of modern volleyball, the first attack, as the initial effective offensive organization following serve reception, is not only a key source of scoring but also reflects a team's tactical proficiency and control over offensive and defensive rhythm (4) modern volleyball, offensive tactics have become increasingly diverse. The proliferation of offensive tactics has transformed the organizational patterns of the first attack. By comparing the changes in first attack tactics across two Olympic Games, we can clearly identify which tactics hold a distinct advantage in high-level competitions.

Volleyball is a continuous sport in which game actions are highly interrelated. Therefore, understanding the connections between different technical actions is essential (5). In volleyball competitions, the effectiveness of attack is influenced by setting, and setting is influenced by reception. These three elements are interlinked; therefore, it is crucial for a team's performance and goals in a match to thoroughly analyze the relationship between reception, setting, and the first attack (6). Previous research has shown that serve reception performance of women's volleyball players has significant differences. As the critical starting point of the first-attack sequence, the quality of serve reception directly affects the effectiveness and type of offensive play. Well-organized serve reception increases the likelihood of structured and effective offensive organization (7). Receiving serve technique, as the foundation of the setter's tactical organization, directly influences the setter's selection of passing attack zones and the effectiveness of their passes. High-quality receiving serve technique provides the setter with optimal passing conditions, thereby enhancing the efficiency of the attack (8). Conversely, if reception techniques are ineffective, the setter cannot effectively distribute the ball, leading to reduced offensive efficiency and potential match loss (9). Research indicates that the quality of setting has become the foundation for attacking tactical choices, particularly evident in fast-attack tactics. Fatahi et al. (10) pointed out that the selection of setting attack zones plays a crucial role in the quality of the attack. Research on the “first-attack” system cannot be conducted in isolation from individual movements but must instead be approached from the perspective of overall coordination to analyze the dynamic relationships between the three movement components. Currently, substantial research has focused on the movement performance of individual technical movements in volleyball matches, while studies on the coordinated relationships between various technical movements remain relatively scarce (11).

Historically, offensive tactics have exhibited distinct characteristics across different eras. Early attacks primarily centered on quick, flat, and fast plays down the middle (6). Modern volleyball offenses have diversified, gradually incorporating multi-point attack patterns such as powerful spikes and back-row attacks. Rule reforms have also propelled the evolution of the first attack. For instance, the introduction of the rally-point system and the libero position has stabilized defense, accelerated the rhythm of games, and consequently highlighted the first attack's pivotal role within.

It is worth noting that the 2020 and 2024 Olympic Games were chosen as study samples because the Olympics represent the highest level of international sports competition and are highly comparable. These two events bring together the world's top women's volleyball teams, whose competitive level and tactical execution represent the highest standards of their time. The four-year interval between the two Olympic Games coincided with a critical period of rapid development in modern volleyball. During this period, the International Volleyball Federation introduced new features in offensive tactics (12), the serve element has been modified (13) and offensive and defensive systems (14), such as increased serve speed, diversified offensive tactics, and improved game strategies, making the stability and efficiency of the first attack system increasingly the core factor in determining the outcome of a match.

This study compares and analyzes the performance of elite women's volleyball teams in various aspects of the first attack during the 2020 Tokyo Olympics and the 2024 Paris Olympics. It reveals the changing trends in the first attack system of elite women's volleyball teams, particularly in receiving serves, setting, and attacking. By combining video analysis, the study identifies the underlying causes of issues within the first attack system and provides an overall understanding of the development trends of the first attack system in elite women's volleyball. The study findings provide a theoretical basis for coaches to develop targeted training programs and optimize tactics, and serve as a reference for elite women's volleyball teams preparing for the 2028 Olympic Games.

## 2 Materials and methods

### 2.1 Sample

This sample includes the women's volleyball competition at the 2020 Tokyo Olympics (held in 2021) and the 2024 Paris Olympics. We selected the deciding sets from each match of the two Olympic Games as the research subjects, observing a total of 64 sets and collecting 2,337 actions of reception, 2,056 actions of set, and 2,118 actions of attack. Sets in free ball situations were not recorded in the attacking zone.

### 2.2 Data collection

Competition footage from two Olympic Games was sourced from publicly accessible platforms such as the official Olympic website, Tencent Video, and CCTV Video. These videos feature high-resolution quality and slow-motion replay capabilities, enabling relatively accurate depiction of technical details during athletes’ serve-receive, passing, and attacking sequences. While publicly broadcast recordings may occasionally omit minor details, the overall image quality and multi-angle coverage ensure the reliability and validity of the study data. Recorded content includes: Reception performance—Reception position—Passing method—Passing attack zone—Attack performance—Attack method. This study was approved by the Academic Committee of school of Athletic Performance, Shanghai University of Sport.

### 2.3 Reliability

The observations were conducted by two observers with specialized training in volleyball. One observer was a nationally certified first-class athlete recognized by the General Administration of Sport of China, and the other was a nationally certified second-class athlete recognized by the General Administration of Sport of China. Before official data collection commenced, a team was randomly selected from a match for preliminary data gathering. To ensure data reliability, a follow-up observation was conducted two weeks later. Data analysis was conducted using SPSS Version 27 for reliability testing. The reliability of the observational data was verified, showing that the inter-observer Cohen's Kappa value was greater than 0.86, indicating that the data are reliable.

### 2.4 Variables

**Figure 1:**
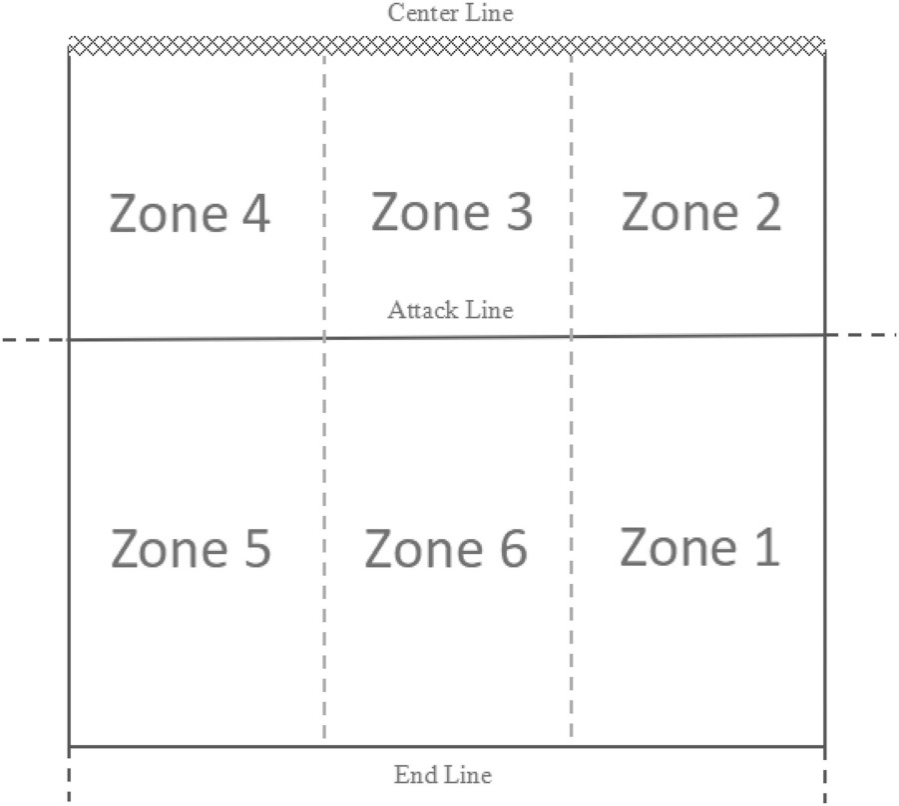
Divisions of volleyball court zones.

Based on the different research topics of previous studies and in conjunction with the main content of this paper, the variables analyzed in this study are as follows:

Regarding the evaluation criteria for receiving serves Qiming Xiao (3) and Barzouka (15), the definitions include the performance of receiving serves and the position of receiving serves. The performance of receiving serves is categorized as follows: Perfect Pass: The first pass has a certain arc, is within 2.5 m of the center line of the court, and is a ball that the setter can handle within two steps of movement. Medium pass: The first pass has a moderate arc, with the second pass near the three-meter line, requiring the setter to have a certain distance to execute the pass. Out-of-system reception: The first pass has no arc or goes directly over the net, requiring the setter to adjust the pass to organize the attack; a pass that goes over the net is also considered an incomplete reception. Error: The ball lands directly in the team's court area or goes out of bounds, resulting in a direct point loss. According to Fatahi et al. (10) the position of the reception is divided into six zones based on the player's position on the court (Figure 1).

The evaluation criteria for setting, as defined by Chen (16) and Barzouka (15), include settings methods and setting attack zones. Setting methods are categorized into bumping, jump setting, back setting, and stationary setting. The setting attack zone refers to the second set after receiving the serve, which is selected on the basis of the positions of players on the court, the opponent's defensive and offensive positions, or the team's offensive tactics; it is divided into six zones based on the positions on the court(Figure 1).

The evaluation criteria for attack tactics, as defined by Zhao (17) and Dou (18), include attack methods and attack performance. Attack methods are categorized as follows: Power Attack: In situations where there is little or no coverage from teammates, the attacker relies primarily on individual strength and skill to break through the opponent's defense or block forcefully, with complete attack movements and strong aggressiveness. Fast attack: A spike is completed quickly near the setter's front or side, primarily executed by the middle blocker. Combination attack: A spike is performed by a back-row player jumping from the attack line while being covered by a front-row player, primarily executed by the back-row outside hitter or middle blocker at positions 1 and 6. Out-of-system attack: When the serve is not properly received, the setter runs a long distance and delivers the ball to the attacker with a high arc, or another player passes the ball to the attacker. The attack is incomplete, lacking aggressiveness, and involves light taps, floaters, or non-attacking plays. Second attack: When the first pass is too high or goes into the net, front-row players can jump and directly spike or float the ball. Tip: This refers to the setter delivering the ball accurately and the attacker intentionally performing a light tap or dropped ball. The performance of the attack is categorized as follows: Scoring: The spike directly scores a point. Blocked: The spike is blocked by the opposing team. General: The spike is defended by the opposing team. Error: The spike goes out of bounds, hits the net, or is completely blocked.

### 2.5 Statistical analyses

This study aimed to test whether the categorical variables had significant differences. The 2020 Tokyo Olympics and the 2024 Paris Olympics were treated as two independent sample groups. Given the relatively limited sample size, the sample data did not fully meet the prerequisites for normal distribution. Therefore, the Mann–Whitney U test, a non-parametric test, was used to test for differences. The statistical significance threshold was set at *p* < 0.05 (significant) and *p* < 0.01 (very significant).

## 3 Results

This section presents a systematic comparison of key technical elements (receiving, passing, and attacking) in the first attack chain of women's volleyball competitions to reveal the characteristics of technical and tactical evolution in the first-attack system of elite women's volleyball teams during two Olympic cycles.

### 3.1 Analysis of changes in reception performance

Regarding the effects of receiving serves, tests showed that there were significant differences in the effectiveness of receiving serves at the Olympics (Table 1). The reception effectiveness of the women's volleyball team showed some differences, but only the “out-of-system reception” effect exhibited significant differences. In OG-20, a total of 1,373 serves were received in decisive sets, while in OG-24, a total of 961 serves were received. In terms of serve reception effectiveness, the percentage decreased from 58.85% in OG-20 to 54.73% in OG-24, though the difference did not reach statistical significance (*p* = 0.16), indicating that there is no significant difference in the effect. In OG-20, 21.85% of returns were “medium pass,” while in OG-24, 21.12% of returns were “medium pass.” Indicating that the “medium pass” effect remained relatively stable and there was no statistically significant difference (*p* = 0.72). The proportion of “out-of-system reception” returns increased from 14.49% in OG-20 to 18.63% in OG-24, and the mean ± standard deviation also increased from 0.14 ± 0.10 to 0.18 ± 0.06, with this change showing a statistically significant difference (*p* = 0.03). The proportion of “errors” in receiving serves increased from 4.81% to 5.52%, and the change in errors was relatively stable, with no significant difference (*p* = 0.72).

**Table 1:** Comparison of receiving serve effectiveness.

Variables	OG-20			OG-24			*p*
	*N*	%	*M* *±* *SD*	*N*	%	*M* *±* *SD*	
Perfect Pass	808	58.85%	0.59 ± 0.14	526	54.73%	0.56 ± 0.15	0.16
Medium pass	300	21.85%	0.22 ± 0.11	203	21.12%	0.21 ± 0.11	0.72
Out-of-system reception	199	14.49%	0.14 ± 0.10	179	18.63%	0.18 ± 0.06	0.03*
Error	66	4.81%	0.05 ± 0.06	53	5.52%	0.05 ± 0.06	0.80
Total	1,373	100%	–	961	100%	–	–

* Indicates 0.01 < *p* < 0.05, indicating a statistically significant difference; **indicates *p* < 0.01, indicating a highly significant difference.

### 3.2 Distribution and trend of reception positions

According to the data analysis in Table 2, In OG-20, the most frequent receiving position was Zone 6 (39.78%). Compared with OG-24, the receiving proportion of Zone 6 showed a slight decrease, with little change in the mean value, and no significant difference was observed (*p* = 0.23). The reception ratio at Zone 1 decreased from 25.02% to 19.54%, which is a significant difference (*p* = 0.01). The reception ratios at zones 2, 3, and 4 decreased from 0.08%, 1.16%, and 0.31% to 0.10%, 0.21%, and 0.13%, respectively. Overall, the proportions were relatively low, indicating that the number of serves received in these three positions was relatively low in both Olympic Games. Among them, Zones 2 (*p* = 0.02) and 3 (*p* = 0.02) showed significant differences with statistical significance; Zone 4 showed no significant difference (*p* = 0.16). Zone 5 also had a relatively high reception ratio in both tournaments, with 31.93% in OG-20 and 41.48% in OG-24, which is a significant difference (*p* < 0.01). This result indicates that Zones 5 and 6 have become the primary target areas for the opposing team's serves.

**Table 2:** Comparison of serve receive zones.

Variables	OG-20			OG-24			*p*
	*N*	%	*M* *±* *SD*	*N*	%	*M* *±* *SD*	
Zone 1	344	25.02%	0.25 ± 0.11	188	19.54%	0.20 ± 0.13	0.01*
Zone 2	11	0.08%	0.01 ± 0.02	1	0.10%	0.01 ± 0.01	0.02*
Zone 3	16	1.16%	0.01 ± 0.03	2	0.21%	0.01 ± 0.01	0.02*
Zone 4	18	1.31%	0.01 ± 0.03	7	0.13%	0.01 ± 0.01	0.16
Zone 5	439	31.93%	0.31 ± 0.14	399	41.48%	0.42 ± 0.17	<0.01**
Zone 6	547	39.78%	0.41 ± 0.14	365	37.94%	0.37 ± 0.14	0.23
Total	1,375	100%	–	962	100%	–	–

* Indicates 0.01 < *p* < 0.05, indicating a statistically significant difference; **indicates *p* < 0.01, indicating a highly significant difference.

### 3.3 Comparison of setting techniques

Table 3 presents the different types of set used by women's volleyball players in volleyball competitions at the two Olympic Games. Overall, the changes in set types remained relatively stable, with only “stationary sets” showing a significant difference (*p* = 0.02). The proportion of stationary sets used in OG-20 was 7.8%, which decreased to 5.13% in OG-24, possibly because this set type limits the team's ability to create offensive combinations. The jump pass had the highest proportion in both Olympic Games, at 49.26% in OG-20 and 50.84% in OG-24, indicating that the jump pass has become the primary setting method in volleyball matches. The mean remained unchanged, and no significant difference was observed (*p* = 0.87), suggesting that the jump pass exhibits high stability in matches. The usage rates of back setting were 31.03% (OG-20) and 31.74% (OG-24), respectively, with relatively high proportions. The average values showed little change, and no significant difference was found (*p* = 0.89). Back sets are an important means of enhancing offensive concealment and multi-point offensive capabilities, and they continue to hold a key tactical position in elite competitions.

**Table 3:** Comparison of setting methods.

Variables	OG-20			OG-24			*p*
	*N*	%	*M* *±* *SD*	*N*	%	*M* *±* *SD*	
Bumping	145	11.9%	0.11 ± 0.10	103	12.29%	0.10 ± 0.08	0.76
Jump setting	600	49.26%	0.44 ± 0.13	426	50.84%	0.44 ± 0.13	0.87
Back setting	378	31.03%	0.27 ± 0.13	266	31.74%	0.29 ± 0.14	0.89
Stationary setting	95	7.8%	0.07 ± 0.07	43	5.13%	0.04 ± 0.05	0.02*
Total	1,218	100%	–	838	100%	–	–

* Indicates 0.01 < *p* < 0.05, indicating a statistically significant difference; **indicates *p* < 0.01, indicating a highly significant difference.

### 3.4 Distribution of set-to-attack zones

Comparing the setting attack zones reveals that in the two Olympic Games (Table 4), the setter passed more frequently to Zones 2 and 4. In OG-20, these passes accounted for 340 times (27.76%) and 535 times (43.67%), respectively. In OG-20, they accounted for 232 (27.68%) and 343 (40.93%), respectively. Their average values remained relatively stable, with no significant differences (*p* = 0.56, *p* = 0.11); Next is the pass to Zone 3, with the proportion of Zone 3 increasing from 14.94% in OG-20 to 17.42% in OG-24, and the average also showing a slight increase, with no significant difference (*p* = 0.23). Zones 5 and 6 had relatively low proportions in both Olympic Games. Zone 5 decreased from 1.71% (*n* = 21) in OG-20 to 0.72% (*n* = 6) in 2024, with a *p*-value of 0.25. Zone 6 saw a slight increase from 5.5% (*n* = 68) to 5.73% (*n* = 48), with a *p*-value of 0.80. The changes in the proportions of these two positions also did not reach statistical significance. Zone 1 had a share of 6.37% (*n* = 78) in OG-20, rising to 7.52% (*n* = 63) by OG-24, with a stable mean and no statistically significant difference (*p* = 0.58).

**Table 4:** Comparison of setting attack zones.

Variables	OG-20			OG-24			*p*
	*N*	%	*M* *±* *SD*	*N*	%	*M* *±* *SD*	
Zone 1	78	6.37%	0.06 ± 0.06	63	7.52%	0.06 ± 0.07	0.58
Zone 2	340	27.76%	0.25 ± 0.11	232	27.68%	0.25 ± 0.13	0.56
Zone 3	183	14.94%	0.13 ± 0.09	146	17.42%	0.15 ± 0.09	0.23
Zone 4	535	43.67%	0.39 ± 0.11	343	40.93%	0.36 ± 0.12	0.11
Zone 5	21	1.71%	0.01 ± 0.05	6	0.72%	0.01 ± 0.02	0.25
Zone 6	68	5.5%	0.05 ± 0.07	48	5.73%	0.05 ± 0.06	0.80
Total	1,225	100%	–	838	100%	–	–

* Indicates 0.01 < *p* < 0.05, indicating a statistically significant difference; **indicates *p* < 0.01, indicating a highly significant difference.

### 3.5 Tactical tendencies in attack types of elite women’s volleyball

According to the data analysis in Table 5, from OG-20 to OG-24, there was some fluctuation in the women's volleyball team's offensive strategies. The proportion of strong attacks in matches increased from 44.81% (OG-20) to 46.25% (OG-24), with the average remaining nearly unchanged (*p* = 0.52, no significant difference); The proportion of quick attacks in matches decreased from 20.05% to 17.88%, with a decrease in the mean value, *p* = 0.26, indicating no significant difference; The proportion of three-dimensional attacks was 10.38% (*n* = 130) at the Tokyo Olympics and 10.03% (*n* = 87) at the OG-24, with the average remaining nearly unchanged, *p* = 0.71, indicating no significant difference; In adjusted attacks, the proportion increased from 12.86% to 13.84%, with a small increase and minimal change in the average, *p* = 0.55, indicating no significant difference; Secondary attacks accounted for the smallest proportion in both Olympic Games and showed a decreasing trend (2.00%, 1.50%), with a slight decrease in the mean, *p* = 0.26, indicating no significant difference; The proportion of spikes was 9.9% (*n* = 124) in OG-20 and 10.50% (*n* = 91) in OG-24, with almost no change in the mean, *p* = 0.72, no significant difference.

**Table 5:** Comparison of attack methods.

Variables	OG-20			OG-24			*p*
	*N*	%	*M* *±* *SD*	*N*	%	*M* *±* *SD*	
Power attack	561	44.81%	0.41 ± 0.16	401	46.25%	0.42 ± 0.12	0.52
Fast attack	251	20.05%	0.19 ± 0.10	155	17.88%	0.16 ± 0.09	0.26
Combination attack	130	10.38%	0.09 ± 0.08	87	10.03%	0.09 ± 0.07	0.71
Out-of-system attack	161	12.86%	0.11 ± 0.08	120	13.84%	0.12 ± 0.08	0.55
Second attack	25	2.00%	0.02 ± 0.03	13	1.50%	0.01 ± 0.02	0.26
Tip	124	9.90%	0.09 ± 0.08	91	10.50%	0.10 ± 0.08	0.72
Total	1,252	100%	–	867	100%	–	–

* Indicates 0.01 < *p* < 0.05, indicating a statistically significant difference; **indicates *p* < 0.01, indicating a highly significant difference.

### 3.6 Comparison of attack performance

A comparative analysis of offensive performance between the 2020 Tokyo Olympics and the 2024 Paris Olympics revealed the following results (Table 6): the total number of attacks in decisive sets for the two Olympics were 1,256 and 867, respectively. In terms of scoring, the OG-20 accounted for 40.13% (*n* = 504), while the OG-24 accounted for 38.52% (*n* = 334), with *p* = 0.41 indicating no significant difference. In the 2020 Games, the direct scoring rate from the first attack by elite women's volleyball teams was the primary method. In terms of “general” performance, the proportion was 36.31% (*n* = 456) in OG-20 and 38.41% (*n* = 333) in OG-24, with a slight increase, indicating no significant difference (*p* = 0.30). In terms of attacks blocked, the proportion was 8.84% (*n* = 111) in OG-20, while in OG-24, it accounted for 8.77% (*n* = 76), with a slight decrease in proportion, *p* = 0.67, indicating no significant difference; the error rate was 14.73% (*n* = 185) in OG-20, and 14.30% (*n* = 124) in Paris, with *p* = 0.56, indicating no significant difference. In this cycle, no statistically significant difference was found in the overall attacking effectiveness of the two Olympic Games.

**Table 6:** Comparison of attack effectiveness.

Variables	OG-20			OG-24			*p*
	*N*	%	*M* *±* *SD*	*N*	%	*M* *±* *SD*	
Scoring	504	40.13%	0.38 ± 0.12	334	38.52%	0.36 ± 0.13	0.41
General	456	36.31%	0.33 ± 0.12	333	38.41%	0.35 ± 0.11	0.30
Blocked	111	8.84%	0.08 ± 0.08	76	8.77%	0.08 ± 0.08	0.67
Error	185	14.73%	0.13 ± 0.08	124	14.30%	0.12 ± 0.08	0.56
Total	1,256	100%	–	867	100%	–	–

* Indicates 0.01 < *p* < 0.05, indicating a statistically significant difference; **indicates *p* < 0.01, indicating a highly significant difference.

## 4 Discussion

This study analyzes the changes in first attack technical indicators in women's volleyball across two Olympic Games, aiming to reveal the evolving trends of the first-attack system in elite women's volleyball. The findings reflect the overall attack style of elite teams and capture the developmental dynamics of the first-attack system. The results also provide a scientific basis for future training, particularly in preparation for the 2028 Olympic Games. The analysis shows that among the components of the first-attack system, serve reception performance and reception zones exhibit significant differences between the two competitions. By contrast, setting and attack remain relatively stable. Moreover, a clear association is found between serve reception quality and the setter's choice of attack zones, which in turn is closely linked to the type of attack executed (5).

Our findings indicate a remarkable increase in the number of serve receptions that failed to reach the optimal setting zone. In 2024, the effectiveness of the “out-of-system reception” return of serve improved remarkably. This trend may be attributed to stronger teams treating the serve as the first line of offense, intensifying their serve power or employing tactical serving strategies to directly disrupt the opponent's reception, particularly when facing stronger teams (13), with the aim of scoring directly. It may also be related to the psychological pressure on the players. During the decisive set, some teams may accelerate the pace of the game, resulting in insufficient preparation time for athletes and errors in receiving serves. Psychological factors also cannot be overlooked. In top-tier competitions like the Olympics, athletes must execute technical maneuvers under intense competition and psychological pressure. Their mental state often affects the stability of the first attack, making it one of the key explanatory variables for the differences observed in this study's findings. Video analysis has shown that ineffective reception can affect the effectiveness of the setter (5). When the reception is not in place during a match, the setter needs to adjust the set to organize the attack (19). Receiving serves plays a fundamental role in the overall tactical system, and the results of a study by Palao et al. (20) indicate a direct correlation between receiving serves and spiking effectiveness. Accurate and semiaccurate receiving serves give setters a wider range of options for organizing attacks and distracting the opponent's defense through tactical plays (7).

The position of receiving the serve is related to the team's rotation position, and this association may be due to the tactical choices made by the opposing team when serving (21). From 2020 to 2024, elite women's volleyball teams tended to serve to Zones 5 and 1 during matches, resulting in receiving positions mainly concentrated in Zones 1 and 5. This tendency causes players in Zones 4 and 1 to take on greater responsibility when receiving serves, preventing attackers from quickly entering an offensive state (22). The study further revealed the interdependent relationship between serving and receiving serves. Barzouka et al. (23) found that opponents primarily used targeted serving or zone serving to disrupt the opposing team's offense. Martínez et al. (24) also discovered correlations between the receiving team's rotation, receiving zone, and attacking performance.

In the two Olympic Games, the women's volleyball team's setting style was relatively stable, mainly dominated by jump setting and back setting, with bumping and stationary setting as auxiliary techniques. The study found that the use of stationary setting has declined, indicating that the women's volleyball team's setting style in competition is evolving toward a more offensive direction. Consistent with the findings of Chen (16), who noted in his study that setters need to increase the speed, power, and concealment of their passes to create favorable conditions for the match, this trend reflects the higher demands placed on passing techniques in terms of match organization efficiency under the backdrop of accelerated game pace and tactical diversification in modern volleyball. The proportion of back passes in the two Olympic Games was basically the same, reflecting that back passes are a highly concealed technique in the game that can distract the opponent's blocking and defense predictions. Tsavdaroglou et al. (22) believe that when the opponent's blocking organization is relatively tight, improving the concealment of passes is an effective way to break through a “high block, high defense” situation. Afonso et al. (8) pointed out that the setting style is not related to offensive effectiveness.

Studies have shown that the setting attack zone is not only related to the position of the attacking player but also depends on the quality of the reception and the team's attacking tactics. For example, the RT1 rotation used in the game is a rotation in which the front row setter receives the ball at Zone 1 and immediately attacks at Zone 2 to reduce the flight time of the ball, interfere with the opponent's block, and improve attacking effectiveness (22). This rotation is relatively difficult to detect during a match. Our study found that in women's volleyball competitions, setters tend to set the ball to Zones 2 and 4 for strong attacks, followed by passing to Zone 3 for fast attacks and to Zone 6 for combination attacks, consistent with the findings of Tsavdaroglou et al. (22). Meanwhile, a study indicates that aggressive attacks significantly increase scoring probability compared to skill-based attacks (23). The increased proportion of adjusted balls and tips in the match may be due to poor reception effectiveness or a tactical response strategy chosen by the attackers, or it may be related to situational factors, for example athletes tend to prefer stable, low-risk technical movements under the pressure of competition (19).

Attacking effectiveness is related to the opponent's block (24). Direct attacking points mainly rely on the attacking team's own strength, effectively identifying and exploiting gaps in the opponent's block to launch powerful attacks, or using techniques such as hitting the ball out of bounds to force the opponent to make defensive mistakes, thereby achieving direct points. A study by Castro et al. (23) indicates that double or triple block can effectively disrupt the attacking team's counterattack system. However, this does not completely eliminate the attacking team's scoring potential, as players can still score directly through powerful spikes and fast-paced attacks during matches. Attacks are generally related to double blocks, and attacking errors may be related to the opponent's strong blocking ability or problems in the coordination between the attacker and the setter. As pointed out by De Conti Teixeira Costa et al. (24), attacking effectiveness is not only related to the attacker but also to the entire process of the attack, with each link interacting and coordinating with each other. This study also reveals that serving technique is closely linked to first-attack performance. High-quality serves often pose significant challenges to the return, thereby reducing the efficiency of the first attack (13), the success or failure of an offensive play depends not only on the individual skills of a single player but more critically on the overall coordination of the team and the seamless collaboration between all positions.

## 5 Conclusions

In elite women's volleyball competitions, the development of the first-attack system shows a trend of “stable foundation and overall coordination.” The number of “out-of-system reception” serves has increased remarkably, reflecting the disruptive effect of serves on the effectiveness of returns in competitions. Opponents use strategies such as targeting specific players or areas to influence the attacker's offensive transition. At the same time, ineffective serve reception can affect the effectiveness of the setter. In women's volleyball competitions, jump setting is the main type of setting, with back setting used as a concealed type of setting to lure the opponent's blocking system. A significant decrease in stationary setting has been observed, indicating that the first attack tactical system in women's volleyball competitions is becoming increasingly complex. In terms of attacking methods and attacking effectiveness, women's volleyball as a whole is becoming more stable. In the entire first-attack system, the effectiveness of receiving serves remains a decisive factor, and the quality of the setter and the passing attack area are the basis of the attacking method. In future training, the focus should be on enhancing athletes’ reception quality and coordination between setters and attackers to optimize the first-attack system further.

## 6 Limitations

Although this study has revealed the developmental trends of the first-attack system in elite women's volleyball competitions, several limitations remain: (1) Limitations of the research sample: This study only selected the decisive sets from two Olympic women's volleyball competitions as analysis samples, which may not fully reflect the changes in the first-attack system throughout the entire competition. (2) Video analysis involves subjectivity. (3) This study primarily focuses on analyzing technical variables related to serving, passing, and spiking and does not include nontechnical variables such as athletes’ physical condition and psychological state.

## Data Availability

The data analyzed in this study is subject to the following licenses/restrictions: the dataset analyzed in this study is available from the first author upon reasonable request. Requests to access these datasets should be directed to mengjuanwang, mengjuanwang0@gmail.com.

## References

[1] Tian MJ. New development in the theoretical system of athletic training in China. J Beijing Univ Phys Ed. (2003) 02:145–8. 10.19582/j.cnki.11-3785/g8.2003.02.001

[2] Wang YX Wang D. Research on use and diagnosis of techniques in technique—oriented racket games. Bull Sport Sci Technol. (2015) 23(04):31–2. CNKI:SUN:SPOR.0.2015-04-018

[3] Xiao QM. Analysis of “first attack” tactics in women’s volleyball in Tokyo olympics based on grey situation decision (Master’s thesis). Beijing Sport University, Beijing (2023).

[4] Zhan JH. A comparative study on the effect of the first attack of China, Japan and Iraq in 2018 and 2019 world men’s (Master’s thesis). Jiangxi Normal University, Nanchang (2020).

[5] González-Silva J Fernández-Echeverría C Conejero M Moreno MP. Characteristics of serve, reception and set that determine the setting efficacy in men’s volleyball. Front Psychol. (2020) 11:222. 10.3389/fpsyg.2020.00222 32132957 PMC7040554

[6] Lobietti R Cabrini P Brunetti M. The side-out complex in volleyball: the effect of reception and attack performance with the final score. In: Hökelmann A Witte K O'Donoghue P, editors. Current Trends in Performance Analysis. World Congress of Performance Analysis in Sport VIII-Proceedings Book. Magdeburg: University of Magdeburg, Shaker Verlag (2009). p. 91–5.

[7] Araújo CRP Tosini L Freire AB Costa GDCT Meira CM Jr. Reception-attack relation in men’s and women’s volleyball during the Rio 2016 Olympics. J Phys Ed Sport. (2020) 20:2008–12. 10.7752/jpes.2020.s3271

[8] Afonso J Mesquita I Marcelino R da Silva JA. Analysis of the setter’s tactical action in high-performance women’s volleyball. Kinesiology. (2010) 42(1):82–9. Available online at: https://hrcak.srce.hr/54245

[9] Rocha ACR Pedrosa GF Freire AB Praça GM Ugrinowitsch H Castro HDO Analysis of the setting and predictive factors of the effect of attack according to game ecology: the case of female volleyball. Kinesiology. (2020) 52(2):217–23. 10.26582/k.52.2.7

[10] Fatahi A Molla RY Drikos S Jadidoleslam S. A comprehensive analysis of the serve reception zone, set zone and attack quality of the top-level volleyball players. Eur J Human Move. (2022) 48:54–63. 10.21134/eurjhm.2022.48.6

[11] Costa GC Caetano RCJ Ferreira NN Junqueira G Afonso J Costa RDP Determinants of attack tactics in youth male elite volleyball. Int J Perform Anal Sport. (2011) 11(1):96–104. 10.1080/24748668.2011.11868532

[12] Lv K Kong C. Analysis of tactical variations in position 3 offense in volleyball matches. Cambridge Sport Sci. (2025) 2025(2):9–16. 10.62852/css/2025/148

[13] Pawlik D Bobula G Mroczek D. The effectiveness and types of serves used in elite women’s and men’s volleyball in the 2021/2022 season. Sci Rep. (2024) 14(1):17286. 10.1038/s41598-024-68262-5 39068291 PMC11283524

[14] Deda N. The study of technical-tactical and physical parameters of modern volleyball through specific models in the defense element. Interdiscip J Res Dev. (2024) 11(2):44. 10.56345/ijrdv11n206

[15] Barzouka K. Comparison and assessment of the setting zone choices by elite male and female volleyball setters in relation to the reception quality. J Phys Ed Sport. (2018) 18:2014. 10.7752/jpes.2018.s5299

[16] Palao JM Manzanares P Ortega E. Techniques used and efficacy of volleyball skills in relation to gender. Int J Perform Anal Sport. (2009) 9(2):281–93. 10.1080/24748668.2009.11868484

[17] Palao JM Lopez-Martinez A Valades D Hernandez E. Manner of execution and efficacy of reception in men’s beach volleyball. Monten J Sports Sci Med. (2019) 8(2):21. 10.26773/mjssm.190903

[18] López E Díez-Vega I Molina JJ. Reception and performance in high level male volleyball: a relational study. J Hum Sport Exerc. (2020) 17(2):409–23. 10.14198/jhse.2022.172.16

[19] Molina-Martín JJ Diez-Vega I López E. Reception-attack transition in volleyball: analysis of spike effectiveness. Apunts Educ Fís Esports. (2022) (149):53–62. 10.5672/apunts.2014-0983.es.(2022/3).149.06

[20] Barzouka K Sotiropoulos K Drikos S Kitsiou A Angelonidis Y. Current trends of the serve skill in relation to the in-game roles of the elite volleyball players: comparison between genders. J Hum Sport Exerc. (2020) 16(2):317–32. 10.14198/jhse.2021.162.08

[21] Martínez EL Martín JJM Bento MSD Vega ID. Spike performance in K1: influence of rotation and reception area on high level men’s volleyball teams. Retos. (2023) 48:213–20. 10.47197/retos.v48.93875

[22] Tsavdaroglou S Sotiropoulos K Barzouka K. Comparison and assessment of the setting zone choices by elite male and female volleyball setters in relation to opposing block organization. J Phys Educ Sport. (2018) 18:2147. 10.7752/jpes.2018.s5325

[23] Castro J Souza A Mesquita I. Attack efficacy in volleyball: elite male teams. Percept Mot Skills. (2011) 113(2):395–408. 10.2466/05.25.PMS.113.5.395-408 22185054

[24] De Conti Teixeira Costa G Afonso J Vieira Barbosa R Coutinho P Mesquita I. Predictors of attack efficacy and attack type in high-level Brazilian women’s volleyball. Kinesiology. (2014) 46(2):242–8. Available online at: https://hrcak.srce.hr/131928

